# Recombinant growth hormone therapy for prepubertal children with idiopathic short stature in Korea: a phase III randomized trial

**DOI:** 10.1007/s40618-017-0786-8

**Published:** 2017-11-04

**Authors:** J. Kim, B.-K. Suh, C. W. Ko, K.-H. Lee, C. H. Shin, J. S. Hwang, H. S. Kim, W. Y. Chung, C. J. Kim, H.-S. Han, N. Y. Kwon, S. Y. Cho, H.-W. Yoo, D.-K. Jin

**Affiliations:** 1 Department of Pediatrics, Samsung Medical Center, Sungkyunkwan University School of Medicine, 81 Irwon-ro, Gangnam-gu, Seoul, 06351 Republic of Korea; 2 Department of Pediatrics, Seoul St. Mary’s Hospital, The Catholic University of Korea, Seoul, Republic of Korea; 3 Department of Pediatrics, Kyungpook National University Hospital, Daegu, Republic of Korea; 4 Department of Pediatrics, Korea University Anam Hospital, Seoul, Republic of Korea; 5 Department of Pediatrics, Seoul National University Children’s Hospital, Seoul, Republic of Korea; 6 Department of Pediatrics, Ajou University Hospital, Suwon, Republic of Korea; 7 Department of Pediatrics, Severance Hospital, Onsei University Health System, Seoul, Republic of Korea; 8 Department of Pediatrics, Inje University Busan Paik Hospital, Busan, Republic of Korea; 9 Department of Pediatrics, Chonnam National University Hospital, Gwangju, Republic of Korea; 10 Department of Pediatrics, Chungbuk National University Hospital, Cheongju, Republic of Korea; 11 Data Management and Clinical Statistics Team, Dong-A ST Co., LTD, Seoul, Republic of Korea; 12 Department of Pediatrics, Medical Genetics Clinic and Laboratory, Asan Medical Center Children’s Hospital, University of Ulsan College of Medicine, 88 Olympic-ro 43-gil, Songpa-gu, Seoul, 05505 Republic of Korea

**Keywords:** Growth hormone, Idiopathic short stature, Clinical trial

## Abstract

**Purpose:**

Several studies have evaluated the effects of growth hormone (GH) on auxological and biochemical parameters in children with non-GH-deficient, idiopathic short stature (ISS). This study evaluated the efficacy and safety of Growtropin^®^-II (recombinant human GH) in Korean patients with ISS.

**Methods:**

This was a 1-year, open-label, multicenter, phase III randomized trial of Growtropin^®^-II in Korean patients with ISS. In total, 70 prepubertal subjects (39 males, 31 females) between 4 and 12 years of age were included in the study. All patients were naive to GH treatment.

**Results:**

Annual height velocity was significantly higher in the treatment group (10.68 ± 1.95 cm/year) than the control group (5.72 ± 1.72, *p* < 0.001). Increases in height and weight standard deviation scores (SDSs) at 26 weeks were 0.63 ± 0.16 and 0.64 ± 0.46, respectively, for the treatment group, and 0.06 ± 0.15 and 0.06 ± 0.28, respectively, for the control group (*p* < 0.001). Serum insulin-like growth factor (IGF-1) and insulin-like growth factor binding protein-3 (IGFBP-3) increased significantly in the treatment group at week 26 compared to baseline. However, the SDS for body mass index (BMI) at 26 weeks did not change significantly in either group. Growtropin^®^-II was well tolerated and safe over 1 year of treatment.

**Conclusions:**

One-year GH treatment for prepubertal children with ISS demonstrated increased annualized velocity, height and weight SDSs, and IGF-1 and IGFBP-3 levels, with a favorable safety profile. Further evaluations are needed to determine the optimal dose, final adult height, and long-term effects of ISS treatment.

## Introduction

Children with idiopathic short stature (ISS) are a heterogeneous group; there are many unidentified causes of short stature, and wide variation in causative biological factors, the extent of bone-age delay, and timing of puberty [1]. ISS is defined as a height more than two standard deviations (SD) below the age-, sex-, and population-matched mean, despite a normal or increased response to growth hormone (GH) on the GH stimulation test, and in the absence of any systemic, endocrine or metabolic disease, chromosome abnormality, or other disease that may compromise growth [2, 3].

GH therapy has been approved by the US Food and Drug Administration (FDA) for patients with GH deficiency (GHD, 1985), chronic renal insufficiency (1993), Turner syndrome (TS, 1997), or Prader-Willi syndrome (PWS, 2000), for children with a history of intrauterine growth restriction (IUGR) [small for gestational age (SGA), 2001], short stature with homeobox-containing gene deficiency (2006), or Noonan syndrome (NS, 2007) [4–6]. In 2003, the FDA extended the indications for GH therapy to include children with ISS who are > 2.25 SD below the mean height, and who are unlikely to catch up in height [7].

Studies evaluating GH therapy in children with ISS for 4–7 years revealed an increase in adult height (AH) of 3–6 cm [1, 3, 8, 9], and a safety profile similar to other GH indications [1]. Although the mean height increased in response to GH therapy, there was significant variability in individual growth responses, including no measurable increase in height standard deviation scores (Ht SDSs) in some patients [9, 10].

The present study was a 1-year, open-label, multi-center, randomized controlled trial (RCT) of recombinant human GH (rhGH) administered to prepubertal Korean children with ISS. We comprehensively analyzed the efficacy and safety of GH therapy.

## Materials and methods

### Subjects

Inclusion criteria for the study were as follows: prepubertal children [evaluated according to the Tanner classification: testicular volume of 4 cc or less (boys) and Tanner stage I breast development (girls)]; height below the third percentile for a Korean population of the same chronological age (CA) and sex; one or more peak GH levels of 10 ng/ml or above, confirmed by GH stimulation testing in those taking two or more of clonidine, glucagon, insulin, and levodopa, and normal karyotype (female); older than 4 years of age with a bone age (BA) of < 11 years (girls) or < 13 years (boys), and a disparity of 3 years or less between BA and chronological age; naive to GH therapy, and; normal thyroid function (or normalized after hormone therapy).

Exclusion criteria were as follows: hypopituitarism, GHD, chronic renal failure, SGA, IUGR, premature birth (gestational age of ≤ 36 weeks), congenital infections, TS, PWS, NS, Russell-Silver syndrome, Seckel syndrome, Down syndrome, Cushing’s syndrome, or other chromosomal abnormalities; congenital or chronic disease; currently receiving a drug that may affect the secretion and action of GH (such as estrogen, androgen, anabolic steroid, corticosteroid, or methylphenidate); and a history of hypersensitivity to GH.

Prior to the screening test, based on consent forms approved by the Korean Ministry of Food and Drug Safety (MFDS, approval no.11886) and the institutional review board of each institution, informed consent was obtained from the subjects themselves and their legally authorized representatives (e.g., parents) by their own discretion.

## Methods

Eligible children were given subcutaneous injections of rhGH at a dose of 1.11 IU (0.37 mg)/kg/week (the standard dose approved by the FDA), divided into six to seven doses per week, for 52 weeks. The rhGH (Growtropin^®^-II, DA-3002) was provided by Dong-A ST Ltd. (Seoul, Korea). Subjects randomized to the treatment group (*n* = 36) received rhGH from week 0 to week 52, while subjects randomized to the control group (*n* = 34) were observed without treatment from week 0 to week 26 and received the rhGH from week 27 to week 52. Randomization was performed at a 1:1 ratio using a block method. Subjects were required to visit their study site at weeks 13, 26, 39 and 52 for measurements of height and weight; assessment of levels of insulin-like growth factor 1 (IGF-1), insulin-like growth factor binding protein 3 (IGFBP-3), and hemoglobin A1C; evaluation of thyroid function; and other laboratory tests. IGF-1 and IGFBP-3 were measured via CLIA using a Siemens Immulite 2000 XPi immunoassay system, and Siemens reagents, in our central laboratory. Subjects also completed questionnaires exploring drug compliance and adverse events, either in person during visits or via telephone between visits (at weeks 6, 19, 32, and 45). Subjects in the control group continued to receive therapy for an extra 6 months at the end of the study, during which they attended a follow-up visit for safety evaluation.

The pubertal stage was evaluated by a pediatric endocrinologist at each visit. BA and anti-GH antibody levels were determined at baseline and at weeks 26 and 52. GH antibody levels were measured with the aid of a GH Ab ELISA Kit (Wuhan EIAAB Science) and an ELx808™ Absorbance Microplate Reader (BioTeck), in our central laboratory. BA was measured by a single pediatric endocrinologist, based on X-rays of the left hand and wrist using the Greulich-Pyle method.

### Outcome variables

The primary efficacy endpoint was the difference in annual height velocity (cm/year) at week 26 between the treatment and control groups. Secondary efficacy endpoints were the differences in Ht SDS, BA, IGF-1, and IGFBP-3 between the treatment and control groups at week 26, the difference in annual height velocity (cm/year) between weeks 0 and 52 (treatment group) and weeks 27 and 52 (control group), and the difference in annual height velocity (cm/year) between weeks 0 and 26 (treatment group) and weeks 0 and 52 (treatment group). The Ht SDS was calculated using the growth standard for Korean children and adolescents. Adverse event rates were summarized by system organ class (SOC) and preferred term (PT) using MedDRA software (ver.17.0; International Federation of Pharmaceutical Manufacturers Associations, http://www.meddra.org). Investigators were instructed to report any adverse clinical or laboratory findings.

### Statistical analysis

Statistical analysis was performed using SAS statistical software (ver. 9.2, SAS Institute, Cary, NC, USA). Data are presented as mean ± SD, as minimum and maximum values for quantitative variables, mean ± SD for efficacy variables, and frequency and percentage for qualitative variables. Descriptive statistics were used to summarize participant baseline characteristics. Efficacy was analyzed using a two-sample *t* test or the Wilcoxon rank sum test, depending on the results of the Shapiro–Wilk normality test; we assessed differences between the control and treatment groups. The Wilcoxon rank sum test was used to evaluate the difference in adherence between the treatment groups of the clinical trial. Safety analysis was performed by reference to both adverse events and laboratory data. Adverse events were summarized descriptively by SOC and PT, as well as by severity and relationship to the study drug. Differences in laboratory values, analyzed separately in normal and abnormal range groups, were determined using McNemar’s test. In cases where the subject dropped out of the study, the annual height velocity (cm/year) was calculated using the height measured at the last visit. A *p* value less than 0.05 was considered to be statistically significant.

## Results

### Patient characteristics and auxological data at baseline

**Fig. 1:**
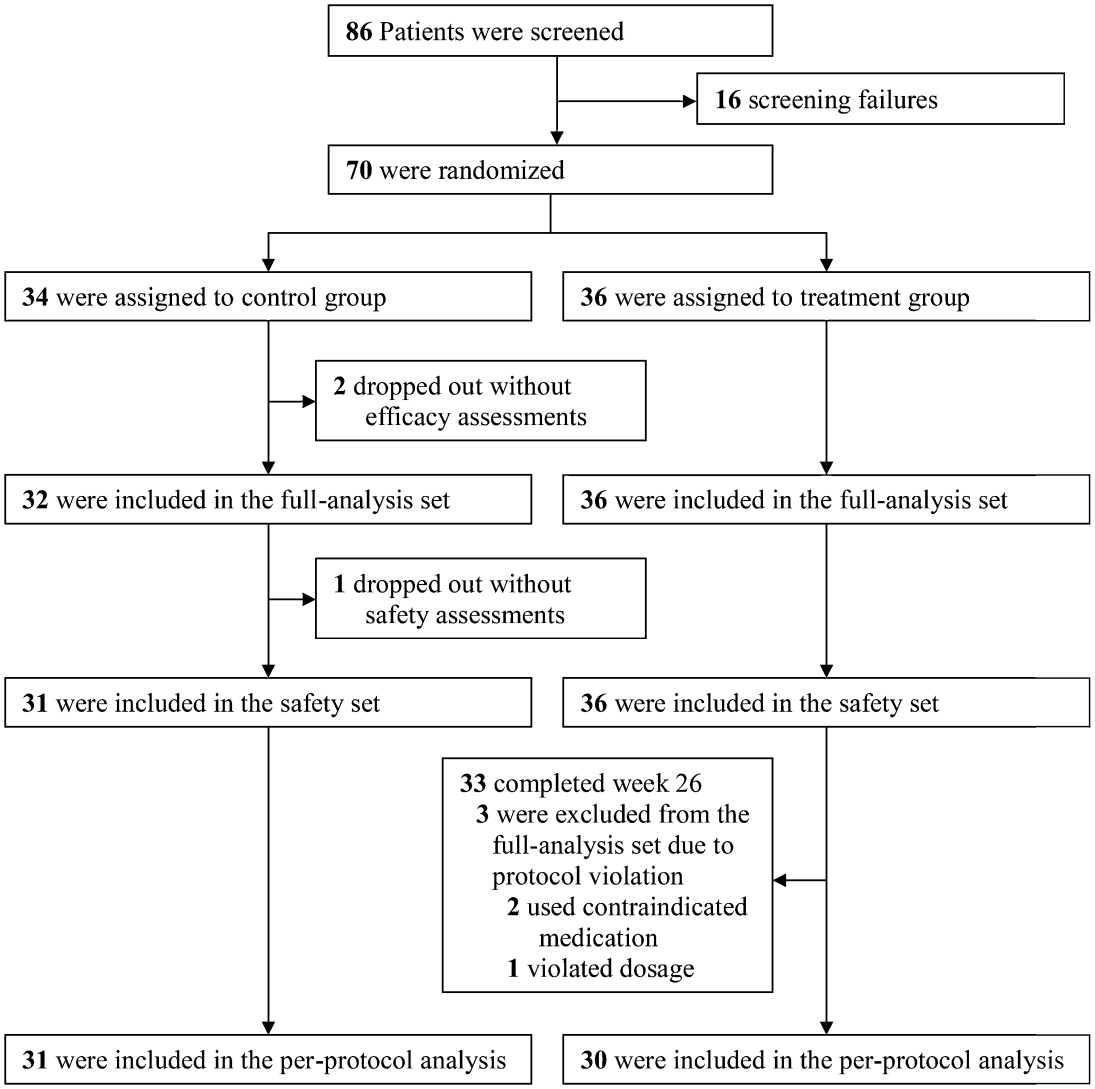
Flow of the distribution of subjects

In total, 86 Korean subjects were recruited from 11 hospitals in February 2012, of whom 70 were enrolled in this study, which ended in April 2014. In total, 34 subjects were randomized to the control group, and 36 to the treatment group. The distribution of subjects is shown in Fig. 1. Of the 70 subjects, there were 39 boys (55.7%) and 31 girls (44.3%); 64 (91.7%) of the 70 subjects completed the 26-week treatment, among whom 63 (90.0%) completed the study. The most common reason for premature discontinuation was ‘withdrawal of consent’ (*n* = 5), followed by ‘protocol violation’ (*n* = 2). The mean chronological age of the subjects was 6.96 ± 2.07 years (range: 4.0–12.1 years). The mean BA was 5.32 ± 2.24 years (range: 2.5–11.00 years). The mean height was 108.31 ± 10.76 cm (-2.37 ± 0.50 SDS), the mean weight was 18.34 ± 4.28 kg (2.03 ± 0.99 SDS), and the mean body mass index (BMI) was 15.45 ± 1.43 (-0.54 ± 0.97 SDS). The pretreatment height velocity did not differ significantly between the two groups. The subjects had no history of GH therapy before this study, and there was no significant group difference in demographic information or baseline characteristics (Table 1).

**Table 1:** Baseline demographics of individuals with ISS

Group	Control (*n* = 34)	Treatment (*n* = 36)	Total (*n* = 70)
Category	*N* (%), mean ± SD (minimum, maximum)		
Male/female	17 (50.0)/17 (50.0)	22 (61.1)/14 (38.9)	39 (55.7)/31 (44.3)
Chronological age (years)	7.17 ± 2.34 (4.3, 12.1)	6.75 ± 1.79 (4.0, 11.0)	6.96 ± 2.07 (4.0, 12.1)
Bone age (years)	5.53 ± 2.62 (2.7, 11.0)	5.11 ± 1.84 (2.5, 11.0)	5.32 ± 2.24 (2.5, 11.0)
Height (cm)	109.18 ± 11.78 (92.87, 133.27)	107.49 ± 9.80 (89.93, 126.17)	108.31 ± 10.76 (89.93, 133.27)
Height SDS^a^	− 2.40 ± 0.40 (− 3.32, − 1.90)	− 2.35 ± 0.58 (− 4.50, − 1.89)	− 2.37 ± 0.50 (− 4.50, − 1.89)
Weight (kg)	18.73 ± 4.39 (13.7, 30.7)	17.97 ± 4.20 (10.5, 27.2)	18.34 ± 4.28 (10.5, 30.7)
Weight SDS^a^	− 1.95 ± 0.76 (− 4.03, − 0.52)	− 2.11 ± 1.17 (− 6.02, − 0.02)	− 2.03 ± 0.99 (− 6.02, − 0.02)
Body mass index (kg/m^2^)	15.55 ± 1.11 (12.65, 17.65)	15.36 ± 1.69 (12.98, 20.71)	15.45 ± 1.43 (12.65, 20.71)
Body mass index SDS^a^	− 0.45 ± 0.93 (− 2.96, 1.19)	− 0.64 ± 1.01 (− 2.53, 2.22)	− 0.54 ± 0.97 (− 2.96, 2.22)
Pretreatment height velocity (cm/year)	5.19 ± 1.17 (2.73, 7.25)	5.41 ± 1.50 (2.73, 8.74)	5.31 ± 1.34 (2.73, 8.74)
Peak GH level (ng/mL)	20.90 ± 14.66 (10.20, 91.00)	21.87 ± 16.88 (10.00, 88.00)	21.40 ± 15.74 (10.00, 91.00)

*GH* growth hormone, *SDS* standard deviation score

^a^Calculated using the modified LMS method suggested by the Korean Center for Disease Control and Prevention (CDC) and the Korean Pediatric Society

### Efficacy results

The full analysis (FA) set was defined as all subjects who received at least one dose of the study drug and had at least one measurement of the primary efficacy endpoint after randomization. The per-protocol (PP) set was defined as all subjects who completed all 26 weeks of the study without any major protocol violation. During the study period, major protocol violation occurred for one subject (2.9%) in the control group and six subjects (16.7%) in the treatment group. The violation types included inclusion/exclusion criteria violation, treatment compliance < 80%, continuous use of contraindicated drugs for ≥ 1 week, and more than three administration and dosage violations. The safety set consisted of 67 subjects (31 in the control group, 36 in the treatment group). The efficacy measures were analyzed in the FA set, which consisted of 68 subjects (32 in the control group and 36 in the treatment group), excluding 2 subjects who did not undergo primary efficacy evaluation after randomization. The PP set included 61 subjects (31 in the control group, 30 in the treatment group).

**Fig. 2:**
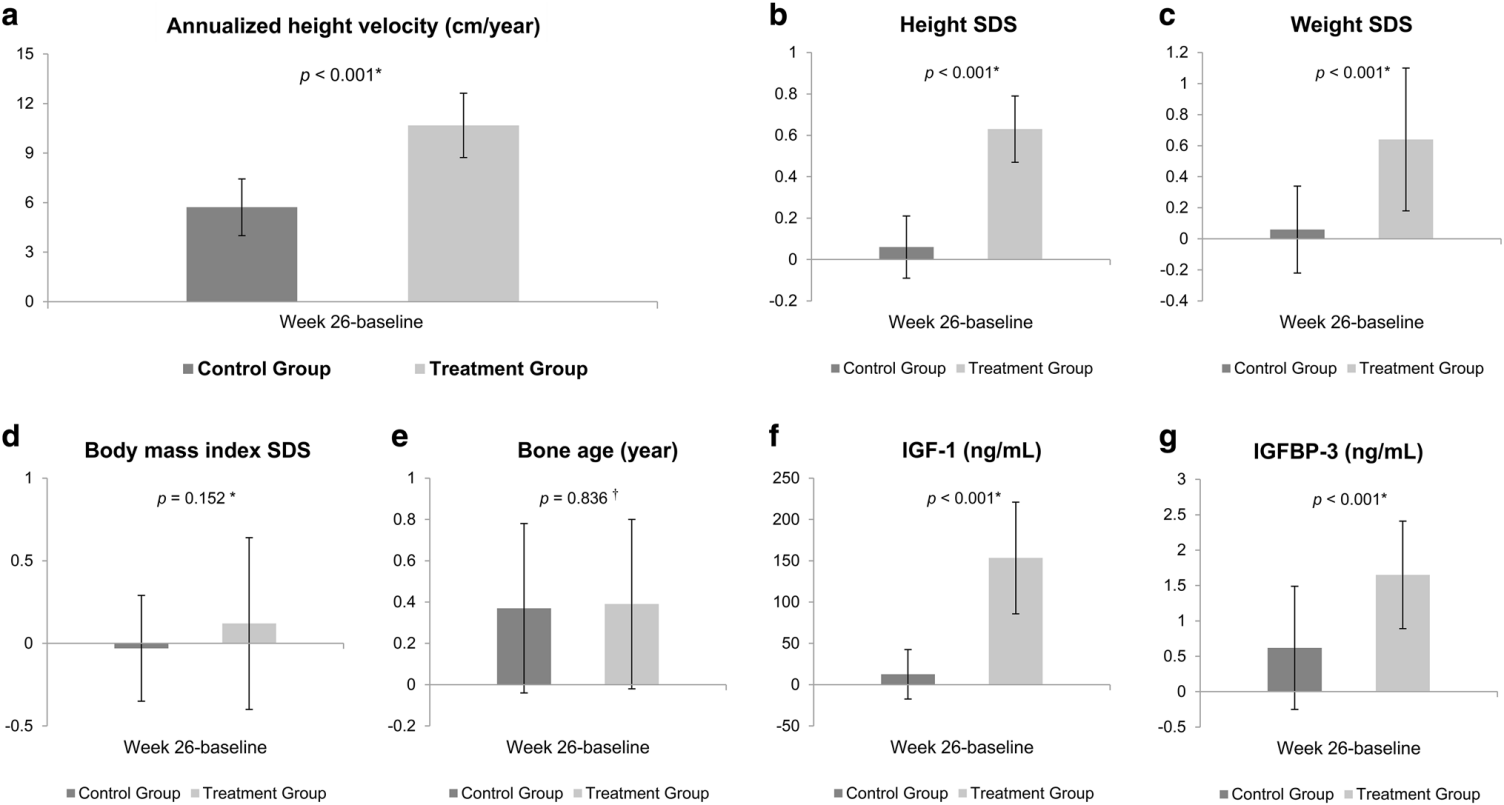
Differences in annualized height velocity, height SDS, weight SDS, body mass index SDS, bone age, IGF-1 level, and IGFBP-3 level at 26 weeks from baseline in the control and treatment groups. **a** annualized height velocity, **b** height SDS, **c** weight SDS, **d** body mass index SDS, **e** bone age, **f** IGF-1 level, **g** IGFBP-3 level. *SDS* standard deviation score, *IGF-1* insulin-like growth factor-1, *IGFBP-3* insulin-like growth factor binding protein-3. *Two-sample *t* test. ^†^Wilcoxon rank sum test

The primary efficacy endpoint, annualized height velocity (cm/year) at week 26, was 5.72 ± 1.72 in the control group and 10.68 ± 1.95 in the treatment group, indicating a statistically significant difference (*p* < 0.001) (Fig. 2, Table 2). Regarding the secondary efficacy endpoints, the Ht SDS increased significantly from the baseline at week 26 in both the control and treatment groups (control group, *p* = 0.033; treatment group, *p* < 0.001). The difference in Ht SDS at week 26 relative to baseline was 0.06 ± 0.15 in the control group and 0.63 ± 0.16 in the treatment group, showing a statistically significant difference between the two groups (*p* < 0.001). These results indicate that short-term GH therapy can enhance growth velocity. Weight SDS at week 26 of the study increased compared to the baseline in both groups and the differences between the two groups were statistically significant (0.64 ± 0.46 in the treatment group and 0.06 ± 0.28 in the control group, *p* < 0.001); however, BMI SDS at week 26 did not change significantly in either group (0.12 ± 0.52 in the treatment group, − 0.03 ± 0.32 in the control group, *p* = 0.152). The BA advanced from week 4 to week 26 in both groups (treatment group, *p* < 0.001; control group, *p* < 0.001). The mean differences between week 4 and week 26 were 0.39 ± 0.41 in the treatment group and 0.37 ± 0.41 in the control group, with no statistically significant difference between the two groups (*p* = 0.836). The BA was not too advanced for CA as the bone maturation rate (change in BA/change in CA) was 0.71 ± 0.79 in the treatment group and 0.74 ± 0.78 in the control group. Serum IGF-1 level in the treatment group increased significantly at week 26 compared to baseline levels (101.16 ± 42.34 ng/mL at baseline vs. 257.61 ± 83.63 ng/mL at week 26; *p* < 0.001). The differences in serum IGF-1 levels were 12.54 ± 29.95 in the control group and 153.34 ± 67.60 in the treatment group, indicating a statistically significant difference between the two groups (*p* < 0.001). Serum IGFBP-3 level also increased significantly at week 26 compared to the baseline (3.23 ± 0.83 ng/mL at baseline vs. 4.89 ± 0.74 ng/mL at week 26; *p* < 0.001). The mean differences in IGFBP-3 between week 4 and week 26 were 0.62 ± 0.87 in the control group and 1.65 ± 0.76 in the treatment group, indicating a statistically significant difference in magnitude of change between the groups (*p* < 0.001).

**Table 2:** Differences in height, height velocity, height SDS, weight SDS, body mass index SDS, bone age, IGF-1, and IGFBP-3 at 26 weeks from baseline in the control and treatment groups

Group	Control (*n* = 32)	Treatment (*n* = 36)	*p* value
Difference in			
Height (cm)			
Baseline	109.24 ± 12.26	107.61 ± 9.69	
Week 26	112.07 ± 11.99	112.87 ± 9.60	
Annualized height velocity (cm/year)	5.72 ± 1.72	10.68 ± 1.95	< 0.001^a^
Height SDS			
Baseline	− 2.39 ± 0.37	− 2.37 ± 0.58	
Week 26	− 2.32 ± 0.43	− 1.75 ± 0.67	
Week 26-baseline	0.06 ± 0.15	0.63 ± 0.16	< .001^a^
*p* value	0.033^c^	< 0.001^c^	
Weight SDS			
Baseline	− 1.98 ± 0.68	− 2.16 ± 1.20	
Week 26	− 1.91 ± 0.70	− 1.47 ± 1.04	
Week 26-baseline	0.06 ± 0.28	0.64 ± 0.46	< 0.001^a^
*p* value	0.228^c^	< 0.001^c^	
Body mass index SDS			
Baseline	− 0.51 ± 0.91	-0.68 ± 1.01	
Week 26	− 0.56 ± 0.89	-0.48 ± 0.90	
Week 26-baseline	− 0.03 ± 0.32	0.12 ± 0.52	0.152^a^
*p* value	0.597^c^	0.177^c^	
Bone Age (year)			
Baseline	5.63 ± 2.66	5.11 ± 1.84	
Week 26	6.06 ± 2.75	5.48 ± 1.91	
Week 26-baseline	0.37 ± 0.41	0.39 ± 0.41	0.836^b^
*p* value	< 0.001^d^	< 0.001^d^	
IGF-1 (ng/mL)			
Baseline	112.44 ± 49.49	101.16 ± 42.34	
Week 26	124.96 ± 54.33	257.61 ± 83.63	
Week 26-baseline	12.54 ± 29.95	153.34 ± 67.60	< 0.001^a^
*p* value	0.027^c^	< 0.001^d^	
IGFBP-3 (ng/mL)			
Baseline	3.29 ± 0.83	3.23 ± 0.83	
Week 26	3.90 ± 0.89	4.89 ± 0.74	
Week 26-baseline	0.62 ± 0.87	1.65 ± 0.76	< 0.001^a^
*p* value	< 0.001^d^	< 0.001^c^	

*SDS* standard deviation score, *IGF-1* insulin-like growth factor-1, *IGFBP-3* insulin-like growth factor binding protein-3

^a^Two sample *t* test

^b^Wilcoxon rank sum test

^c^Paired *t* test

^d^Wilcoxon signed rank test

The annual height velocity values were 10.48 ± 1.70 (cm/year) after weeks 27–52 in the control group and 10.17 ± 1.23 (cm/year) after weeks 0–52 in the treatment group, showing no statistically significant difference (*p* = 0.423) (Table 3). The difference in annual height velocity in the treatment group, at week 52 versus week 26, was -0.78 ± 1.03 cm/year, which was a statistically significant difference (*p* < 0.001) despite being less than 1 cm/year. Analysis with the PP set revealed efficacy results similar to those of the FA set (data not shown).

**Table 3:** Change in annual height velocity from week 0 to week 52 in the treatment and control groups

Group	Control (*n* = 32)	Treatment (*n* = 36)	*p* value
Height (cm)			
Week 0	109.24 ± 12.26	107.61 ± 9.69	
Week 26	112.45 ± 11.99	112.79 ± 9.45	
Week 52	117.79 ± 11.88	117.33 ± 9.54	
Annualized height velocity (cm/year) at week 26 and week 52 in the treatment group			
Week 0–26		10.93 ± 1.64	
Week 0–52		10.17 ± 1.23	
*p* value		< 0.001^a^	
Annual height velocity at week 52 in the treatment group, and after weeks 27–52 in the control group			
Week 27–52/week 0–52	10.48 ± 1.70	10.17 ± 1.23	0.423^b^

^a^Paired *t* test was used for within-group comparisons

^b^Two sample *t* test was used for between-group comparisons

### Safety results

The follow-up set for additional safety evaluation at the end of the study consisted of 30 control group subjects only. The compliance rate was 97.65 ± 3.69% in the 26 weeks after the non-treatment period in the control group and 92.60 ± 21.00% over the 52 weeks in the treatment group. We found no statistically significant difference between the two groups (*p* = 0.446). One adverse drug reaction (ADR) occurred in one subject in the treatment group, while none occurred in the control group. The reported ADR was a mild rash. Three serious adverse events (SAEs) occurred in three different subjects. In the control group, one subject underwent an ophthalmologic operation for correction of strabismus during a non-treatment period. In the treatment group, one subject developed acute pharyngotonsillitis, and hypertrophy of the adenoids was observed in another subject. However, none of these events was related to the drug and all three subjects recovered without sequelae.

Among the 67 subjects, 21 (67.7%) children in the control group reported 71 adverse events and 25 (69.4%) children in the treatment group reported 78 adverse events (Table 4). The most common adverse event was infection, which was reported in 18 (58.1%) children in the control group and 17 (47.2%) children in the treatment group. Upper respiratory tract infection, which was a frequently occurring event among the study population, comprised more than half of the adverse events. All remaining adverse events were mild.

**Table 4:** Adverse events by system organ class (weeks 0–52)

System organ class	Number of subjects (%) [number of events]	
	Control (*n* = 31)	Treatment (*n* = 36)
Blood and lymphatic system disorders		1 (2.8) [1]
Eye disorders		1 (2.8) [1]
Gastrointestinal disorders	5 (16.1) [7]	5 (13.9) [8]
General disorders and administration site conditions	2 (6.5) [2]	5 (13.9) [5]
Immune system disorders	1 (3.2) [1]	3 (8.3) [3]
Infections and infestations	18 (58.1) [56]	17 (47.2) [50]
Injury, poisoning and procedural complications	3 (9.7) [3]	
Investigations		1 (2.8) [1]
Nervous system disorders	1 (3.2) [1]	1 (2.8) [1]
Respiratory, thoracic and mediastinal disorders		3 (8.3) [5]
Skin and subcutaneous tissue disorders		3 (8.3) [3]
Surgical and medical procedures	1 (3.2) [1]	

No serious ADRs were reported and there was no withdrawal due to adverse events. As for the clinical laboratory findings regarding blood chemistry and thyroid function, most of the parameters, except phosphorus level, did not show statistically significant within-group changes and were classified as changes from ‘outside the normal range’ to ‘normal’ (Table 5). Phosphorus levels were found to be equal to or close to the upper limit of the normal range, and were considered to not be clinically meaningful. The increases in anti-GH level at 26 weeks from baseline were 0.21 ± 0.54 ng/mL in the control group and 0.30 ± 0.35 ng/mL in the treatment group, with no statistically significant difference between the two groups (*p* = 0.658). Vital signs and concomitant medication did not show statistically significant differences between the groups. The safety results of the control and treatment groups, during the 26 weeks and at follow-up, were similar.

**Table 5:** Laboratory results at baseline and week 26 in the treatment and control groups

		Control (*n* = 31)		Treatment (*n* = 36)	
		*N*	Mean ± SD	*N*	Mean ± SD
ALP (IU/L)	Baseline	31	282.3 ± 179.53	36	308.6 ± 187.27
	Week 26	29	305.7 ± 197.19	32	357.0 ± 221.52
Cholesterol (mg/dL)	Baseline	31	166.7 ± 27.01	36	178.0 ± 29.07
	Week 26	29	169.9 ± 27.38	32	172.1 ± 25.26
Triglyceride (mg/dL)	Baseline	31	88.48 ± 40.26	36	94.03 ± 60.10
	Week 26	29	100.3 ± 53.51	31	112.8 ± 73.35
Hemoglobin A1c (%)	Baseline	31	5.35 ± 0.30	36	5.30 ± 0.26
	Week 26	29	5.37 ± 0.27	32	5.40 ± 0.23
TSH (mIU/L)	Baseline	31	3.02 ± 1.53	36	3.01 ± 1.54
	Week 26	31	2.70 ± 1.59	33	2.66 ± 2.16
Free thyroxine (ng/dL)	Baseline	31	1.29 ± 0.21	36	1.33 ± 0.23
	Week 26	31	1.29 ± 0.18	33	1.37 ± 0.33
Anti-hGH antibody	Baseline	31	0.29 ± 0.43	36	0.24 ± 0.15
	Week 26	31	0.50 ± 0.44	33	0.51 ± 0.34

*ALP* alkaline phosphatase, *hGH* human growth hormone, *TSH* thyroid stimulating hormone

## Discussion

In this study, GH therapy applied to Korean children with ISS resulted in a significant increase in annual height velocity (10.68 ± 1.95 cm/year) and mean Ht SDS (0.63 ± 0.16) after 6 months. In addition, the annual increase in height was 10.17 ± 1.95 cm/year during the first year. No clinically relevant differences, from baseline to the end of the trial, were observed between the treatment and control groups on physical examination of vital signs, BA, or laboratory test results.

The efficacy results of this study are similar to those of previous studies [8, 11, 12]. The previous studies showed significantly increased growth velocity in the first year of treatment (7–9 cm/year, compared to 4–5 cm/year before treatment), and a difference in height velocity between treatment and control (or placebo) groups of approximately 2 cm/year [11]. The mean Ht SDS of the treatment group also exceeded that of the control group, by 0.60 SDS [8].

One short-term, uncontrolled study of Korean children with ISS treated with Eutropin^®^ (Korean product, LG Chem.) was published in 2014 [12]. Compared to the cited study, we enrolled approximately twice as many subjects, and analyzed the efficacy and safety of GH therapy over a longer treatment period.

Several studies suggested that long-term GH treatment increases adult height [1, 3, 13]. It has already been reported that GH therapy using rhGH, such as Humatrope^®^ (Eli Lilly) [14] or Genotropin^®^ (Pfizer) [15], increases final height in children with ISS. Previous reports suggest that the height outcome in ISS depends on the age at the start of the treatment and baseline Ht SDS [16, 17]. Children with ISS may have a short stature due to reduced sensitivity to GH, despite a normal GH concentration, and inherently defective sensitivity to GH may be ameliorated by administering GH at a dose higher than the recommended dose for GHD. Wit et al. suggested that the GH dose chosen for the first year of therapy has a significant impact on the final Ht SDS, with a dose of 0.37 mg/kg/week being more effective than a dose of 0.24 mg/kg/week [18]. In a recent study, the 2-year efficacy of GH therapy demonstrated that high- or standard-dose GH treatment groups grew to within the normal range (mean Ht SDS, –1.4); overall, most of the subjects (83%) reached the normal range [19]. Therefore, early treatment of GH with supraphysiological doses appears to produce the greatest increases in adult height [15, 18].

In this study, the safety profile for the doses of GH employed during 1 year was generally consistent with what would be expected for a pediatric population: only well-known adverse events associated with GH treatment occurred, and no new safety concerns emerged [20, 21]. In evaluating the growth response of the treatment group, it was reassuring that the GH treatment with the FDA-approved dose for ISS had a good safety profile [22]. Mean blood glucose and glycosylated hemoglobin levels did not change during treatment, and there was no case of diabetes in either group. Therefore, the influence of GH on carbohydrate metabolism was similar to that found in previous studies on patients with ISS [14, 18, 23]. There was a significant difference in serum IGF-I levels between the treatment and control groups; however, increased IGF-1 levels were within the normal range using age-appropriate reference standards. Previous studies did not find any evidence that GH treatment using the FDA-approved doses for ISS influences the onset of puberty, or enhances pubertal development [24]. The change in mean BA was similar in male and female subjects, across all groups, throughout this trial. The safety outcomes of both the control and treatment groups, during both the 26-week study period and follow-up, were similar to those of previous studies [3, 20]. The long-term safety of GH has not been clearly shown. The Safety and Appropriateness of Growth Hormone Treatments in Europe (SAGhE) study found that mortality increased in adults treated with rhGH as children, particularly in those who had received more than 50 μg/kg/day of GH; mortality caused by bone tumors or cerebral hemorrhage was of most concern [22]. However, the GH dosage of the present study did not exceed 50 μg/kg/day and we encountered no short-term mortality (1 year), and no bone tumor or cerebral hemorrhage. Additional, longer follow-up studies are needed.

In conclusion, the analysis undertaken in this study demonstrates that GH treatment can increase the height velocity and Ht SDS of children with ISS, and there was no significant difference in safety between the treatment and control groups. Further studies should be conducted to determine the optimal criteria for ISS treatment, and to evaluate efficacy and safety over the long term.
